# Pediatric Trauma Trends Before and After Recreational Cannabis Legalization in Nevada: A Retrospective Repeated Cross-Sectional Study

**DOI:** 10.3390/children13050681

**Published:** 2026-05-15

**Authors:** Jenna Serr, Vidhani Goel, Roberto Sagaribay, Kavita Batra, Ami P. Shah

**Affiliations:** 1 Pediatric Emergency Medicine, Kirk Kerkorian School of Medicine at UNLV, Las Vegas, NV 89102, USA; jenna.serr@unlv.edu; 2 Office of Research, Kirk Kerkorian School of Medicine at UNLV, Las Vegas, NV 89106, USA; vidhani.goel@unlv.edu (V.G.); roberto.sagaribay@unlv.edu (R.S.); 3 School of Public Health, University of Nevada, Las Vegas, NV 89154, USA; 4 Department of Medical Education, Kirk Kerkorian School of Medicine at UNLV, Las Vegas, NV 89154, USA; 5 Pediatric Emergency Medicine, University Medical Center of Southern Nevada, Las Vegas, NV 89102, USA

**Keywords:** pediatric trauma, cannabis legalization, motor vehicle collisions, health care utilization, socioeconomic disparities

## Abstract

**Highlights:**

**What are the main findings?**

- Overall pediatric trauma incidence remained stable, with post-legalization shifts toward more motor vehicle-related injuries and fewer pedestrian injuries.
- ICU utilization patterns changed, with fewer admissions but longer ICU lengths of stay, alongside shifts in socioeconomic and insurance profiles.

**What are the implications of the main findings?**

- Cannabis legalization was not associated with increased pediatric trauma rates, but may be linked to changes in injury patterns and severity.
- Ongoing surveillance and targeted, equity-focused injury prevention strategies are needed, particularly for motor vehicle-related injuries.

**Abstract:**

**Background**: Cannabis legalization has raised concerns regarding its potential influence on injury patterns, particularly among children. However, evidence on pediatric trauma remains limited. **Objective**: To examine trends in pediatric trauma incidence, injury mechanisms, healthcare utilization, and socioeconomic characteristics before and after recreational cannabis legalization in Nevada. **Methods**: A retrospective repeated cross-sectional study of trauma registry data was conducted using pediatric trauma activations recorded between 2013 and 2023. Incidence rates per 100,000 population were calculated using census data. Pre-legalization (2013–2016) and post-legalization (2017–2023) periods were compared using incidence rate ratios (IRRs) and bivariate tests. Socioeconomic status was assessed using the Distressed Communities Index (DCI). **Results**: Among 1772 pediatric trauma activations, overall incidence remained stable (21.6 vs. 21.4 per 100,000; IRR = 0.99, 95% CI: 0.90–1.09). Post-legalization, motor vehicle collision-related injuries increased (49.3% vs. 41.3%, *p* = 0.002), while pedestrian injuries declined (25.5% vs. 32.4%). ICU admissions decreased (19.5% vs. 27.3%, *p* < 0.001), although ICU length of stay increased (5.9 vs. 4.0 days, *p* = 0.005). A higher proportion of patients originated from less distressed communities post-legalization (*p* = 0.021), alongside shifts in insurance coverage (*p* < 0.001). **Conclusions**: Pediatric trauma incidence remained stable following cannabis legalization in Nevada; however, shifts in injury mechanisms, healthcare utilization, and socioeconomic patterns were observed. Because cannabis legalization was assessed at the population level and individual cannabis exposure was not directly measured, findings should be interpreted as temporal associations rather than causal effects. These findings highlight the need for ongoing surveillance and targeted, equity-focused injury prevention strategies.

## 1. Introduction

Trauma continues to be the leading cause of morbidity and mortality in the pediatric population after one year of age, and particularly in the teenage years [1,2,3]. Research has established that the use of psychoactive substances decreases sensory perception, increases reaction times, and affects coordination, thus increasing the risk of injury and motor vehicle collisions [4,5,6,7,8,9]. From a motor vehicle collision (MVC) perspective, a positive correlation between alcohol use and impairment in driving has been well documented. Accordingly, various safety laws are in place regarding Driving Under the Influence (DUI) [10]. After alcohol, cannabis is the most commonly detected substance in MVCs.

Among pediatric trauma mechanisms, MVCs, pedestrian injuries, and bicycle-related incidents remain major contributors to morbidity and mortality, particularly among school-aged children and adolescents [1,2,3]. Prior literature has demonstrated that impaired driving and risky transportation behaviors substantially increase the risk of MVC-related injuries [4,5,6,7,8,9,10]. Given the established association between psychoactive substance use and impaired driving performance, the present study primarily focuses on motor vehicle-related pediatric trauma patterns in the context of cannabis legalization.

As of November 2025, apart from the legalization of medical use of cannabis, its use for recreational purposes has been legalized by 24 states and District of Columbia (DC) [11]. The state of Nevada legalized cannabis for medical use on 1 July 2010, while recreational cannabis legalization, including implementation of legal retail sales, began on 1 January 2017 [12]. Accordingly, the present study defined the post-legalization period as 1 January 2017, through 31 December 2023. From a public health and safety perspective, DUI laws need to address driving under the influence of cannabis (DUIC). From a public health and safety perspective, DUI laws need to address driving under the influence of cannabis (DUIC).

The impact of cannabis use and legalization remains unclear and controversial. Research on the subject is challenging for various reasons, including differences in state laws and a lack of reliable testing that correlates with cannabis intoxication, given the varied effects based on the mode and duration of consumption by the individual [4,5,6,7]. A further confounding factor includes the effect of cannabis when combined with other psychoactive substances [10,11,12,13,14,15].

While research in the adult population is limited, most studies have focused on fatal MVC outcomes and have yielded mixed results [16,17]. Initial research indicated that medical marijuana laws may be somewhat protective and translate into fewer, or at least no significant change in the rate of fatal MVCs [2,18,19]. However, this has not consistently held true over time [20,21]. Prior studies have shown that cannabis use increases the risk of fatal MVCs [21,22,23,24] and non-traffic-related injuries in the adult population [25] without a significant change in pedestrian-related fatal MVCs [2]. Reports also indicate that cannabis use is associated with higher rates of healthcare utilization [24,26,27], although some studies have not observed statistically significant increases over longer follow-up periods [28].

In contrast, research evaluating pediatric populations remains limited. In the pediatric age range, younger children may be affected due to driver impairment, while adolescents are more likely to experiment with substances and engage in risky behaviors [16,19,20,21,22,23,24,25,26,27,28,29,30,31,32]. The majority of existing research in the United States involves fatal crash data and focuses on alcohol-related impairment [33,34,35], with limited evaluation of non-fatal crashes [36] or cannabis-specific effects [14,25,37]. To date, alcohol use by drivers transporting children has been identified as a major risk factor for child endangerment [13,36,38], but comparable evidence for cannabis remains sparse [14]. Evidence regarding non-fatal trauma, injury mechanisms, and healthcare utilization in pediatric populations is particularly lacking. Accordingly, the present findings provide additional observational evidence regarding pediatric trauma patterns in a real-world policy environment following cannabis legalization.

Given these gaps, evaluating real-world pediatric trauma trends in the context of cannabis legalization is critical for informing injury prevention strategies and public health policy. The purpose of this study was to (1) evaluate the incidence of pediatric trauma activations at a Level I trauma center and teaching hospital in the Southwestern United States before and after legalization of cannabis; (2) analyze healthcare resource utilization, including hospital length of stay, ICU admission, ICU length of stay, ventilator use, ventilator days, and hospital disposition; (3) examine in-hospital mortality; and (4) assess socioeconomic factors at both individual and community levels. The study hypothesis proposed that there would be no change in MVC-related pediatric trauma activations before and after recreational cannabis legalization at the study site.

## 2. Methods

### 2.1. Study Design and Setting

A retrospective repeated cross-sectional study of trauma registry data from a Level I trauma center at a teaching hospital in the Southwestern United States was conducted. The study period spanned 1 January 2013 to 31 December 2023, encompassing pre-legalization (2013–2016) and post-legalization (2017–2023) periods relative to recreational cannabis legalization in Nevada, with the post-legalization period beginning 1 January 2017, corresponding to implementation of legal recreational cannabis retail sales. Throughout the study period, the institution consistently functioned as the primary Level I trauma center in the region, and trauma registry data were collected using standardized institutional trauma registry coding and trauma activation practices.

### 2.2. Study Population

The study included pediatric patients aged 0–18 years who presented with trauma activations related to motor vehicle collisions (MVCs), including pedestrian and bicycle incidents involving a motor vehicle, during the study period. Patients were identified using the institutional trauma registry database. Patients were included if they met trauma activation criteria and had a diagnosis code consistent with MVC-related injury. Patients with non-MVC-related mechanisms of injury or those who did not meet trauma activation criteria were excluded.

### 2.3. Variables and Measures

Study variables included exposure, outcomes, and covariates related to demographic, clinical, and socioeconomic characteristics. The primary exposure was the time period relative to recreational cannabis legalization in Nevada, categorized as the pre-legalization period (2013–2016) and post-legalization period (2017–2023). The post-legalization period corresponded to implementation of legal recreational cannabis retail sales beginning 1 January 2017. The primary outcome was the annual incidence rate of pediatric trauma activations per 100,000 pediatric population. Secondary outcomes included injury characteristics (mechanism, location, severity, and probability of survival), healthcare utilization measures (hospital admission, ICU admission, ICU length of stay, ventilator use, ventilator days, and hospital disposition), and mortality outcomes (emergency department and inpatient mortality). Demographic variables included age, sex, race, and ethnicity. Injury severity was assessed using the Abbreviated Injury Scale (AIS) and Injury Severity Score (ISS), as recorded in the trauma registry. Socioeconomic characteristics were evaluated using the Distressed Communities Index (DCI), a validated zip code-level composite socioeconomic measure incorporating indicators of educational attainment, employment, poverty, median income, housing vacancy, and business establishment growth. Patients were assigned DCI categories based on residential zip code information available in the trauma registry and classified as prosperous, comfortable, mid-tier, at-risk, or distressed communities according to established DCI criteria [39]. Insurance payer type was also assessed. Detailed definitions and measurements of all variables are summarized in Table 1.

### 2.4. Ethical Considerations

This study was conducted in accordance with institutional guidelines and was reviewed by the Institutional Review Board. Given the retrospective nature of the study and use of de-identified data, informed consent was waived.

### 2.5. Statistical Analysis

Annual incidence rates were calculated by dividing the number of pediatric trauma activations each year by the corresponding pediatric population and expressed per 100,000 population. Because the pre- and post-legalization periods differed in duration, annualized population-adjusted incidence rates and incidence rate ratios (IRRs) were used to facilitate time-adjusted comparisons between periods rather than relying on cumulative case counts alone. Incidence rate ratios (IRRs) with 95% confidence intervals (CIs) were used to compare pre-legalization (2013–2016) and post-legalization (2017–2023) periods.

Descriptive statistics were used to summarize patient characteristics and outcomes. Categorical variables were reported as frequencies and percentages and compared using chi-square tests. Continuous variables were summarized as means with standard deviations and compared using independent-sample *t*-tests or analysis of variance (ANOVA), as appropriate.

Assumptions for statistical tests were evaluated prior to analysis. Normality of continuous variables was assessed using graphical methods and the Shapiro–Wilk test, and homogeneity of variances was evaluated using Levene’s test. Where assumptions were not met, appropriate alternative methods or transformations were considered. A two-sided *p*-value of <0.05 was considered statistically significant. All analyses were conducted using IBM SPSS Statistics version 29.0 (IBM Corp., Armonk, NY, USA). This study was reported in accordance with the Strengthening the Reporting of Observational Studies in Epidemiology (STROBE) guidelines for observational studies [40].

## 3. Results

### 3.1. Study Population Characteristics

A total of 1772 pediatric trauma activations were included in the analysis, with 629 cases during the 4-year pre-legalization period and 1143 cases during the 7-year post-legalization period. To account for unequal observation periods, incidence comparisons were based on annualized population-adjusted rates and incidence rate ratios. There were no statistically significant differences in age, sex, or ethnicity between the two periods. However, the distribution of race differed significantly (*p* = 0.002), with a higher proportion of Black or African American patients in the post-legalization period (Table 2).

### 3.2. Incidence of Pediatric Trauma

Annual incidence rates of pediatric trauma activations remained relatively stable over the study period. The incidence rate was 21.6 per 100,000 in the pre-legalization period and 21.4 per 100,000 in the post-legalization period, with no statistically significant difference (IRR = 0.99, 95% CI: 0.90–1.09) (Table 3 and Table 4).

### 3.3. Injury Characteristics

Significant changes in injury mechanisms were observed between periods (*p* = 0.002). The proportion of motor vehicle collision-related injuries increased from 41.3% pre-legalization to 49.3% post-legalization, while pedestrian injuries decreased from 32.4% to 25.5% (Table 5). No statistically significant differences were observed in injury location or mean Injury Severity Score (ISS). However, a higher proportion of patients in the post-legalization period had a greater predicted probability of survival (*p* < 0.001).

### 3.4. Healthcare Utilization

Hospital admission from the emergency department decreased in the post-legalization period (75.5% vs. 70.2%, *p* = 0.016). ICU admissions also declined significantly (27.3% vs. 19.5%, *p* < 0.001). Despite fewer ICU admissions, the mean ICU length of stay increased from 4.0 to 5.9 days (*p* = 0.005). No significant differences were observed in ventilator use or ventilator days (Table 6).

### 3.5. Mortality Outcomes

Mortality remained low across both periods. There were no statistically significant differences in emergency department mortality or inpatient mortality between pre- and post-legalization periods (Table 7).

### 3.6. Socioeconomic Characteristics

Significant differences in socioeconomic characteristics were observed. The distribution of the Distressed Communities Index (DCI) shifted post-legalization (*p* = 0.021), with a higher proportion of patients from more prosperous communities and fewer from distressed communities (Table 8). Insurance coverage also changed significantly, with increases in both Medicaid/government and private insurance coverage and a marked decrease in uninsured or self-pay patients (*p* < 0.001).

## 4. Discussion

This study evaluated population-level trends in pediatric trauma following recreational cannabis legalization in Nevada and found that overall incidence remained stable over time. This finding remained consistent even after accounting for changes in the pediatric population, suggesting that legalization was not associated with a measurable increase in pediatric trauma activations or life-threatening injuries. However, important shifts were observed in injury mechanisms, healthcare utilization, and socioeconomic characteristics. These findings contribute to the evolving literature examining pediatric injury epidemiology in the context of changing cannabis policies and broader societal shifts.

The finding of stable pediatric trauma incidence aligns with several prior studies in adult populations that reported no statistically significant changes in emergency department visits or fatal crash rates following cannabis legalization [2,13,20,21]. However, the existing literature remains mixed and continues to evolve, particularly regarding pediatric and non-fatal trauma outcomes. Some studies have demonstrated increased prevalence of cannabis use among drivers involved in fatal motor vehicle collisions [23], while others have reported increased odds of motor vehicle collisions associated with driving under the influence of cannabis [20,41], although not consistently across all analyses [42,43]. These inconsistencies likely reflect differences in study design, measurement of exposure, and confounding factors.

Despite stable incidence, significant shifts in injury mechanisms and healthcare utilization patterns were observed during the post-legalization period. Several factors may explain these findings beyond cannabis legalization alone. Importantly, a substantial portion of the post-legalization study period (2020–2023) overlapped with the COVID-19 pandemic, which significantly altered mobility patterns, traffic density, pedestrian activity, healthcare-seeking behavior, and injury exposure among pediatric populations. During the COVID-19 pandemic, changes in school attendance, recreational activities, mobility patterns, transportation behaviors, and healthcare-seeking practices were widely reported and may have contributed to shifts in pediatric trauma exposure and healthcare utilization [44,45,46,47,48,49]. Several studies have additionally demonstrated substantial reductions in pediatric emergency department visits and emergency hospital admissions during the COVID-19 pandemic, likely reflecting both reduced injury exposure and avoidance of healthcare services for minor and even serious conditions [45,46,47,48,49]. Travel restrictions, lockdown measures, school closures, and implementation of remote learning may also have contributed to the observed decline in pediatric injuries and changes in healthcare utilization patterns during this period [45,46,47,48,49]. Prior studies have similarly demonstrated substantial changes in pediatric trauma epidemiology during the COVID-19 era, including altered injury mechanisms, reduced trauma activations, shifts in injury severity, and changes in healthcare utilization patterns [45,46,47,48,49]. Because the COVID-19 pandemic overlapped substantially with the post-legalization study period, pandemic-related behavioral and healthcare system disruptions may represent an important source of temporal confounding when interpreting observed trends.

These pandemic-related societal and healthcare disruptions may partially explain several patterns identified in this study. These include stable overall trauma incidence, increased MVC-related injuries, fewer pedestrian injuries, reduced hospital and ICU admissions, and higher predicted probabilities of survival during the post-legalization period. Additionally, evolving trauma triage practices and healthcare system adaptations during the pandemic may have influenced admission thresholds and trauma activation patterns. Additional environmental and urban design factors may also have contributed to changes in injury mechanisms. These factors include pedestrian infrastructure, roadway configuration, traffic density, transportation patterns, driving behaviors, and traffic safety enforcement. Because the post-legalization period included a longer observation window than the pre-legalization period, cumulative injury counts should be interpreted cautiously. However, comparisons of injury mechanisms in this study were based primarily on proportional distributions within each period rather than raw counts alone, thereby reducing the influence of unequal observation duration on prevalence estimates. These contextual variables were not directly measured in the current study and should be explored in future research. Because these contextual factors, including the COVID-19 pandemic, occurred concurrently with recreational cannabis legalization, and individual-level cannabis exposure or intoxication data were not available, the findings should be interpreted cautiously as temporal associations rather than direct causal effects attributable solely to legalization. Future longitudinal and multicenter studies are needed to better disentangle the independent effects of cannabis legalization from broader societal and pandemic-related influences on pediatric trauma trends.

Changes in healthcare utilization were also identified. Both hospital and ICU admissions decreased in the post-legalization period, while ICU length of stay increased. This pattern is consistent with national trends [49] and may reflect evolving triage practices, including updates to national field triage guidelines and American College of Surgeons (ACS) criteria [50,51], which have raised concerns regarding potential under-triage of pediatric patients [52]. Longer ICU stays during the post-legalization period may reflect multiple factors, including evolving clinical practices, operational factors, or changes in ICU admission and discharge patterns over time. Notably, ventilator use and mortality remained stable, suggesting no substantial change in overall injury severity.

Significant shifts were also identified in socioeconomic characteristics. A higher proportion of patients originated from more prosperous communities, while fewer were from distressed areas, and insurance coverage patterns shifted toward increased Medicaid/government and private insurance utilization. These findings may reflect broader demographic and policy changes, including population shifts and healthcare coverage expansion associated with the Affordable Care Act [53]. Given the well-established relationship between socioeconomic disadvantage and childhood injury risk [54], these changes highlight the need for continued attention to equity in injury prevention and trauma care.

Cannabis use has been well documented to impair neurocognitive function, including attention, reaction time, and executive functioning, all of which are critical for safe driving [6,7,15,38,55]. Meta-analyses have demonstrated increased risk of motor vehicle collisions among cannabis-impaired drivers, with pooled odds ratios ranging from modest to moderate increases depending on study design and adjustment for confounding factors [56,57,58,59]. However, the present study did not include individual-level data on cannabis use or driver impairment. Therefore, findings should be interpreted as temporal associations rather than causal effects of cannabis legalization, as multiple factors, including polysubstance use, behavioral changes, and policy shifts may influence observed outcomes.

Recent pediatric injury epidemiology studies have emphasized the importance of monitoring evolving injury mechanisms and healthcare utilization trends among children and adolescents in response to changing societal and policy environments [46,47,49]. From a public health perspective, these findings underscore the importance of continued surveillance of pediatric trauma patterns in the context of evolving recreational cannabis policies. Prevention strategies should focus on high-risk mechanisms, particularly MVC-related injuries, and incorporate education for adolescents and caregivers regarding impaired driving. Additionally, addressing socioeconomic disparities remains critical for reducing the burden of pediatric injury.

### Strengths and Limitations

This study has several strengths. It utilizes a large trauma registry dataset spanning a decade, allowing for longitudinal assessment of pediatric trauma trends before and after cannabis legalization. The inclusion of detailed clinical variables, injury characteristics, and healthcare utilization measures provides a comprehensive evaluation of trauma patterns. Additionally, the integration of community-level socioeconomic indicators, such as the Distressed Communities Index, offers important context for understanding disparities in pediatric injury.

However, several limitations should be considered. As a retrospective repeated cross-sectional study, causal relationships between cannabis legalization and observed outcomes cannot be established. The absence of individual-level data on cannabis use, intoxication status, driving under the influence of cannabis (DUIC), or polysubstance exposure limits the ability to directly attribute observed changes to cannabis-related impairment or establish causal relationships between recreational cannabis legalization and pediatric trauma outcomes. Residual confounding may persist due to unmeasured or incompletely measured factors, including behavioral, environmental, and policy-related variables. In addition, contextual confounding is likely, as the post-legalization period coincided with major external influences, including the COVID-19 pandemic, changes in mobility patterns, and updates to trauma triage guidelines. The overlap between the post-legalization period and the COVID-19 pandemic may have substantially influenced observed temporal trends through changes in traffic patterns, mobility, healthcare utilization, school attendance, recreational activity, and trauma exposure independent of cannabis legalization. Broader societal and environmental factors, including changes in transportation patterns, traffic density, healthcare-seeking behaviors, healthcare utilization, urban mobility, school closures, and post-pandemic behavioral shifts, may also have contributed to temporal changes in pediatric trauma patterns independent of cannabis legalization. These concurrent influences should be considered when interpreting the observed associations. These factors may have independently affected injury mechanisms, healthcare utilization, and trauma activation practices. Future studies using interrupted time-series analyses or sensitivity analyses excluding pandemic years may help better isolate the independent association between recreational cannabis legalization and pediatric trauma trends. Although the study institution consistently functioned as the primary Level I trauma center in the region throughout the study period, overall hospital referral patterns remained relatively stable. However, evolving trauma triage recommendations, potential operational shifts in trauma activation practices over time, regional population growth, and demographic changes in Southern Nevada may also have contributed to temporal variations in pediatric trauma incidence and healthcare utilization.

Socioeconomic status was assessed at the community level using zip code–based measures, which may not fully capture individual-level variability and could introduce ecological bias. Finally, as a single-center study conducted at a Level I trauma center, the findings may have limited generalizability to other regions with different demographic, policy, or healthcare system characteristics.

## 5. Conclusions

Recreational cannabis legalization in Nevada was not associated with a significant change in the overall incidence of pediatric trauma activations. However, shifts in injury mechanisms, healthcare utilization, and socioeconomic patterns were observed. These results highlight the complex interplay between policy, behavioral factors, and social context and underscore the need for continued surveillance and targeted, equity-focused injury prevention strategies in pediatric populations.

## Figures and Tables

**Table 1:** Definitions and Measurement of Study Variables.

Category	Variable	Definition/Measurement	Type
Exposure	Legalization period	Categorized based on timing of cannabis legalization: pre-legalization (2013–2016) and post-legalization (2017–2023)	Categorical
Primary Outcome	Pediatric trauma incidence	Annual number of trauma activations divided by pediatric population, expressed per 100,000 children	Continuous (rate)
Injury Characteristics	Injury mechanism	Categorized as motor vehicle collision (MVC), pedestrian injury, or bicycle/motorized vehicle-related injury	Categorical
	Injury location	Classified by anatomical region (e.g., head/neck, thorax, abdomen, musculoskeletal, multiple sites)	Categorical
	Injury severity	Assessed using Abbreviated Injury Scale (AIS) and Injury Severity Score (ISS)	Continuous/Ordinal
	Probability of survival	Derived from trauma registry (categorized as higher probability, preventable death, non-preventable death)	Categorical
Healthcare Utilization	Hospital admission	Admission from emergency department (yes/no)	Binary
	ICU admission	Admission to intensive care unit (yes/no)	Binary
	ICU length of stay	Number of days in ICU	Continuous
	Ventilator use	Use of mechanical ventilation (yes/no)	Binary
	Ventilator days	Duration of ventilator support in days	Continuous
	Hospital disposition	Discharge outcome (home, morgue, other)	Categorical
Mortality Outcomes	ED mortality	Death occurring in the emergency department (yes/no)	Binary
	Inpatient mortality	Death occurring during hospitalization (yes/no)	Binary
Demographic Variables	Age	Age at time of injury (years)	Continuous
	Sex	Male or female	Categorical
	Race	Self-reported race categories	Categorical
	Ethnicity	Hispanic or non-Hispanic	Categorical
Socioeconomic Variables	Distressed Communities Index (DCI)	Validated zip code–level composite socioeconomic index based on indicators of education, employment, poverty, income, housing vacancy, and business growth; categorized as prosperous, comfortable, mid-tier, at-risk, or distressed	Categorical
	Insurance payer type	Classified as Medicaid/government, private/commercial, or other (self-pay/unknown)	Categorical

**Table 2:** Baseline Characteristics of the Sample During Pre- and Post-Legalization Periods (N = 1772).

Variables	Overall			Pre-Legalization 1 January 2013–16 January 2017		Post-Legalization 17 January 2017–31 December 2023		*p*-Value	Effect Size	Test Statistic
	*n*	%	95% *CI*	*n*	%	*n*	%			
Gender										
Female	684	38.6	36.3, 40.9	256	40.7	428	37.4	0.178	0.032	1.813
Male	1088	61.4	59.1, 63.7	373	59.3	715	62.6			
Race										
White	716	40.4	38.1, 42.7	275	43.7	441	38.6	0.002	0.091	14.766
Black or African American	410	23.1	23.1, 25.2	115	18.3	295	25.8			
Asian or Pacific Islander	71	4	3.1, 5.0	31	4.9	40	3.5			
American Indian/Alaskan Native or Others *	575	32.4	30.3, 34.7	208	33.1	367	32.1			
Ethnicity										
Hispanic	569	32.1	29.9, 34.3	192	31.6	377	33.3	0.465	−0.018	0.535
Non-Hispanic	1171	66.1	63.8, 68.3	416	68.4	755	66.7			
	*M*	*SD*	*95% CI*	*M*	*SD*	*M*	*SD*	*p-value*	*Point Estimate*	*95% CI*
Age at Injury	10.79	4.4	10.6, 11	10.6	4.5	10.9	4.4	0.102	−0.081	−0.793, 0.071

**Table 3:** Annual Pediatric Trauma Activations and Incidence Rates per 100,000 Pediatric Population in Nevada, 2013–2023 (N = 1772).

Year	Trauma Activations (*n*)	Incidence Rate (Per 100,000 People)	95% CI (LCL, UCL)
2013	164	23.1	19.8, 26.9
2014	159	22.2	19, 26
2015	159	21.6	18.5, 25.2
2016	147	19.7	16.7, 23.1
2017	139	18.5	15.7, 21.8
2018	156	20.4	17.5, 23.9
2019	139	18.0	15.2, 21.1
2020	163	20.9	17.9, 24.3
2021	176	23.2	20, 26.9
2022	180	23.7	20.5, 27.5
2023	190	24.9	21.6, 28.7

Absolute annual trauma activation counts are presented to facilitate interpretation of year-to-year fluctuations alongside population-adjusted incidence rates. All rates are calculated per 100,000 people unless otherwise specified.

**Table 4:** Pediatric Trauma Activation Incidence Rates and Rate Ratios by Recreational Cannabis Legalization Period (N = 1772).

Legalization Period	Total Ped Population	Trauma Activation	Incidence Rate	95% CI (LCL, UCL)	Incidence Rate Ratio	95% CI (LCL, UCL)
Pre	2,912,669	629	21.6	20, 23.4	0.99	0.898, 1.091
Post	5,349,520	1143	21.4	20.1, 22.6		

All rates are calculated per 100,000 people unless otherwise specified.

**Table 5:** Comparison of Injury Characteristics Among Pediatric Trauma Patients Before and After Recreational Cannabis Legalization (N = 1772).

	Pre-Legalization (*n*, %)	Post-Legalization (*n*, %)	*p*-Value	Overall Effect Size	Test Statistic
Injury Mechanism					
Bicycle, Motorcycle, or Motorized Scooter	165 (26.2)	288 (25.2)	0.002	0.084	12.523
Motor Vehicle Collision	260 (41.3)	563 (49.3)			
Pedestrian	204 (32.4)	292 (25.5)			
Injury Location					
Abdominopelvic	41 (6.5)	49 (4.3)	0.348	0.056	5.588
External	161 (25.6)	274 (24)			
Head/Neck	124 (19.7)	239 (20.9)			
Multiple Sites	151 (24)	286 (25)			
Musculoskeletal	134 (21.3)	265 (23.2)			
Thorax	18 (2.9)	30 (2.6)			
Highest AIS Score					
Minor	181 (28.8)	350 (30.6)	0.032	0.083	12.199
Moderate	199 (31.6)	366 (32)			
Serious	143 (22.7)	283 (24.8)			
Severe	75 (11.9)	82 (7.2)			
Critical	29 (4.6)	60 (5.2)			
Untreatable/Potentially Unsurvivable	2 (0.3)	2 (0.2)			
Injury Severity Score					
Very Minor Injury	162 (25.8)	298 (26.1)	0.746	0.033	1.946
Mild Injury	189 (30)	364 (31.8)			
Major Injury	150 (23.8)	276 (24.1)			
Critical or Very Severe Injury	126 (20)	203 (17.8)			
Fatal or Currently Untreatable Injury	2 (0.3)	2 (0.2)			
Probability of Survival					
Higher Probability	594 (96.7)	1010 (98.7)	<0.001	0.098	15.859
Possibility of Preventable Death	9 (1.5)	0 (0)			
Death May Be Non-Preventable	11 (1.8)	13 (1.3)			
	M (SD)	M (SD)	*p*-value	Cohen’s d	t
Injury Severity Score	9.8 (11.2)	9.2 (10.3)	0.200	0.060	1.2

* *p*-values < 0.05 are considered statistically significant.

**Table 6:** Hospital Disposition and Critical Care Utilization by Legalization Period (N = 1772).

	Variable	Pre-Legalization *n* (%)	Post-Legalization *n* (%)	*p*-Value	Overall Effect Size	Test Statistic
Final Disposition						
	Home	581 (92.4)	1052 (92)	0.429	0.031	1.691
	Morgue	20 (3.2)	28 (2.4)			
	Other	28 (4.5)	63 (5.5)			
Admitted from ED						
	Yes	475 (75.5)	802 (70.2)	0.016	0.057	5.770
	No	154 (24.5)	341 (29.8)			
Admitted to ICU						
	Yes	172 (27.3)	223 (19.5)	<0.001	0.090	14.378
	No	457 (72.7)	920 (80.5)			
Ventilator						
	Yes	67 (10.7)	120 (10.5)	0.920	0.002	0.010
	No	592 (89.3)	1023 (89.5)			
		M (SD)	M (SD)	*p*-value	Cohen’s d	t
ICU Days		4 (6.5)	5.9 (7)	0.005	−0.281	−2.793
Ventilator Days		5.6 (8.5)	7.1 (7.9)	0.206	−0.194	−1.246

* *p*-values < 0.05 are considered statistically significant.

**Table 7:** Emergency Department and Inpatient Mortality by Legalization Period (N = 1772).

Outcome	Pre-Legalization *n* (%)	Post-Legalization *n* (%)	*p*-Value
Emergency Department Mortality			
Yes	8 (1.3)	15 (1.3)	0.943
No	621 (98.7)	1128 (98.7)	
Inpatient Mortality			
Yes	12 (1.9)	13 (1.1)	0.188
No	617 (98.1)	1130 (98.9)	

**Table 8:** Socioeconomic Indicators by Legalization Period (N = 1772).

		Pre-Legalization (*n*, %)	Post-Legalization (*n*, %)	*p*-Value	Overall Effect Size	Test Statistic
Distressed Communities Index (DCI)						
	Prosperous	135 (22.3)	301 (27.4)	0.021	0.082	11.500
	Comfortable	71 (11.7)	138 (12.5)			
	Mid-Tier	62 (10.2)	109 (9.9)			
	At Risk	183 (30.2)	340 (30.9)			
	Distressed	155 (25.6)	212 (19.3)			
Payer Source						
	Medicaid and Other Government	259 (41.2)	632 (55.3)	<0.001	0.375	248.882
	Private/Commercial Insurance	174 (27.7)	463 (40.5)			
	Other (Self-Pay, Unknown)	196 (31.2)	48 (4.2)			

* *p*-values < 0.05 are considered statistically significant. DCI: Validated zip code-level composite socioeconomic index based on indicators of education, employment, poverty, income, housing vacancy, and business growth; categorized as prosperous, comfortable, mid-tier, at-risk, or distressed.

## Data Availability

The data used in this study are not publicly available due to institutional and ethical restrictions related to patient privacy and are not available for sharing.

## Abbreviations

ACS	American College of Surgeons
AIS	Abbreviated Injury Scale
CI	Confidence Interval
DCI	Distressed Communities Index
DUI	Driving Under the Influence
DUIC	Driving Under the Influence of Cannabis
ED	Emergency Department
ICU	Intensive Care Unit
IRR	Incidence Rate Ratio
ISS	Injury Severity Score
MVC	Motor Vehicle Collision
THC	Tetrahydrocannabinol
